# Current Perspectives for Treating Adolescents with Obesity and Type 2 Diabetes: A Review

**DOI:** 10.3390/nu16234084

**Published:** 2024-11-27

**Authors:** Elżbieta Niechciał, Paulina Wais, Jan Bajtek, Andrzej Kędzia

**Affiliations:** Department of Pediatric Diabetes, Clinical Auxology and Obesity, Poznan University of Medical Sciences, 60-572 Poznan, Polandjan.bajtek@gmail.com (J.B.);

**Keywords:** obesity, weight gain, type 2 diabetes, adolescents, weight management, pharmacotherapy, pharmacological treatment, metabolic surgery

## Abstract

**Background**: Childhood obesity is an epidemic and a significant health concern all over the world. Several factors can influence excess weight gain, including eating behaviors, physical inactivity, and genetics. Children and adolescents with obesity have a four-times greater risk of developing type 2 diabetes (T2D) compared with their normal-weight peers. The management of obesity before the development of its comorbidities may prevent its escalation into significant medical and psychosocial problems. However, treatment options for obesity and T2D in youth remained limited for many years, and moreover, available drugs were characterized by low efficacy. The Treatment Options for Type 2 Diabetes in Adolescents and Youth (TODAY) study showed that metformin in monotherapy failed in almost 52% of children with T2D, while adjuncts to rosiglitazone and lifestyle intervention failed in 38.6% and 46.6%, respectively. Recently approved antiobesity medications and/or bariatric surgery are revolutionizing the management of adolescents with obesity and T2D. This work aims to provide a comprehensive overview of the current treatment possibilities for childhood obesity and T2D. **Methods**: An in-depth review of articles with evidence-based research from different countries discussing novel management options for adolescents with obesity and/or T2D was conducted in this review paper. **Results**: The new medications, such as SGLT2 receptor agonists and GLP-1 agonists, are highly effective in treating T2D in adolescents with obesity. **Conclusions**: Based on the performed literature review, the recent approval of a novel generation of drugs seems to be the dawn of a new era in childhood obesity and T2D treatment.

## 1. Introduction

Obesity is the most prevalent nutritional disorder among children and adolescents. In recent decades, the prevalence of childhood obesity increased dramatically, reaching epidemic dimensions. According to the World Health Organization (WHO), over 340 million youth aged 5–19 years and almost 40 million children under the age of 5 years suffered from overweight or obesity in 2016 and 2020, respectively [1,2]. The occurrence of obesity in children and adolescents is increasing in many low-income and middle-income countries, while in high-income countries, it has plateaued, usually at high levels [3]. Nowadays, the WHO and national and international scientific societies recognize obesity as a chronic progressive disease [2,4,5,6,7]. Several factors can lead to excess weight gain, such as poor eating habits, physical inactivity, and unhealthy sleeping manners. Health-related risk behaviors, genetic background, and obesogenic environment also are vital factors. As the prevalence of obesity grows, so does the prevalence of associated comorbidities, including prediabetes and type 2 diabetes (T2D) [8]. Adolescents with obesity face four times the risk of developing T2D than those with a body weight within the normal range [9]. However, trends in T2D occurrence in youth with obesity vary considerably due to different degrees of obesity, age range of the sampled population, or racial/ethnic variation [10].

Abnormal glucose metabolism in individuals with obesity results from peripheral and hepatic insulin resistance followed by a progressive deterioration in beta-cell function. The relationship between obesity and T2D is even stronger in adolescents than in adults. Data from the SEARCH for Diabetes in Youth (SEARCH) study demonstrated a high prevalence of T2D in adolescents with excess weight. In accordance, T2D developed in 79.4% and 10.4% of children with obesity and overweight, respectively [11]. Moreover, higher BMI during adolescence is linked to a significant risk of T2D development in adulthood. Interestingly, the pathogenesis of T2D in youth is similar to T2D in adults; however, youth-onset T2D is associated with lower insulin sensitivity, insulin hypersecretion, and more rapid loss of beta-cell function [12,13,14]. The Treatment Options for Type 2 Diabetes in Adolescents and Youth (TODAY) study demonstrated through studies with hyperglycemic clamp methodology and oral glucose tolerance tests (OGTTs) that in adolescents with T2D beta-cell function declined relatively quickly, about 20–35% per year [14]. This is in contrast to adults with T2D, in which loss of beta-cell function was reported at 7–11% per year [13]. In addition, early-onset T2D is linked to significantly higher mortality, more T2D complications, and risk of adverse cardiovascular events compared to type 1 diabetes [15]. In the TODAY study, microalbuminuria was found in 6.3% of participants at the beginning of the study, while at the end of follow-up, it was already observed in 16.6% of adolescents. The cumulative incidence of T2D-related comorbidities such as nephropathy was 54.8%, and neuropathy and retinopathy were 32.4% and 51%, respectively, at the end of the study, with an average duration of T2D being 13.3 ± 1.8 years [12].

Finally, recent evidence has shown that the stigmatization of adolescents with obesity is a relatively common problem. Young people very often experience victimization and bullying, which altogether may lead to binge-eating disorders, decreased physical activity, and even isolation from society and loneliness [4,5,16,17]. Then, obesity and T2D are conditions that can significantly reduce life expectancy, negatively affect quality of life, and raise healthcare expenditure. This highlights the significance of early intervention in attaining better weight control and preventing the frequency of obesity and T2D complications in youth [4,17]. This review provides a comprehensive overview of recently published evidence-based studies on novel agents and surgical options for childhood obesity and T2D management.

## 2. Materials and Methods

This review is narrative, and no systematic literature search was performed; each author identified and critically reviewed the most relevant papers. The work presents several evidence-based studies on recently approved drugs by the U.S. Food and Drug Administration (FDA) and the European Medicines Agency (EMA) for treating adolescents with obesity and T2D and bariatric surgeries performed in pediatric populations. The following electronic databases were searched for relevant full-text articles in the English language: PubMed, Google Scholar, Scopus, EMBASE, and Web of Science. The search period was from May 2023 to July 2024 using the following keywords: obesity; type 2 diabetes; obesity-induced type 2 diabetes; childhood obesity; children; adolescents; youth; complications; comorbidities; nephropathy; neuropathy; retinopathy; weight management; treatment; management; regimen; guidelines; nonpharmacological therapy; lifestyle interventions; nutritional modification; behavioral intervention; physical activity; pharmacotherapy; pharmacological agents; antidiabetic medications; anti-obesity medications drugs; metformin; 1,1-dimethylbiguanide; insulin; glucagon-like peptide-1 receptor agonists; liraglutide; exenatide; dulaglutide; semaglutide; orlistat; phentermine; topiramate; sodium-glucose co-transporter-2 inhibitor; empagliflozin; dapagliflozin; approval; the U.S. Food and Drug Administration; the European Medicines Agency; effectiveness; sides effects; bariatric surgery; weight-loss surgery. All articles published between January 2001 and July 2024 were checked by title, abstract, and full text. Reviews, guidelines, and experimental studies (in vitro/ex vivo or animal studies) were excluded. As a result, 11 studies were selected, including 1 large study on bariatric surgery and 6 and 4 studies regarding new-generation drugs currently approved for use in youth with T2D and obesity, respectively. It aimed to detect the most clinically significant papers related to the topic and provide a theoretical point of view, considered a valuable educational tool in continuing medical education.

## 3. Treatment Options for T2D and Obesity in Children and Adolescents

### 3.1. Nonpharmacologic Therapy

Lifestyle interventions, including nutritional and behavioral modifications and enhancing physical activities, are a cornerstone of managing childhood obesity and T2D [4,5,17]. The main goal in changing lifestyle behaviors is to promote body mass reduction and, consequently, reduce insulin resistance. It has been reported that decreases in BMI of 0.5 kg/m^2^ or more lead to improvements in insulin sensitivity [18]. International societies such as the American Diabetes Association (ADA) and International Society for Pediatric and Adolescent Diabetes (ISPAD) advise that young individuals with obesity and/or T2D should at least have 60 min of moderate to vigorous physical activity daily [6,7]. The nutrition of youth with obesity should be focused on personalized nutrition approaches that ensure adequate nutritional content tailored to physiological conditions, ages, and sex-specific needs. Dietary components such as ultraprocessed foods and sugar-sweetened beverages should be eliminated from the diet at the baseline in individuals with obesity [6]. However, lifestyle intervention studies in youth with obesity provide conflicting data. Some studies, including behavioral components, showed substantial weight reduction compared to typical management or passive control, with long-term interventions having the greatest benefit [19,20,21]. Conversely, the TODAY study demonstrated no difference in weight loss between the group receiving intensive lifestyle interventions in conjunction with metformin and the group treated with metformin only [12,14]. Moreover, the benefits observed in randomized clinical trials might be difficult to translate to real-world settings.

### 3.2. Metformin

Metformin (1,1-dimethylbiguanide) is one of the oldest antidiabetic medications, which is used as the first-line therapy for the treatment of T2D in metabolically stable children aged 10 years and older, as adjuncts to diet and exercise and sometimes in combination with other glucose-lowering agents [6,7]. For many years, metformin, along with insulin, were the only drugs approved for treating T2D in youth. Metformin is a complex medication that acts through several molecular mechanisms. It differs from other antihyperglycemic agents because of its unique mechanisms of action. Primarily, it reduces hepatic glucose production and intestinal absorption of glucose and decreases insulin resistance by increasing peripheral glucose uptake and utilization [22].

It is predominantly used as a medication for managing T2D, although it has also been studied for its effects on lipid profiles in various populations, including youth. Research has indicated that metformin can have a positive effect on lipid profiles, such as reducing total cholesterol, low-density lipoprotein (LDL) cholesterol, and triglycerides while potentially increasing high-density lipoprotein (HDL) cholesterol [23,24,25]. Moreover, it has been described that metformin may have a beneficial effect on the normalization of ovulatory abnormalities in girls with polycystic ovary syndrome (PCOS), which affects around 20% of girls with T2D [24,25]. While data are limited for children, studies conducted in adult populations have shown that metformin also protects against cardiovascular disease [26]. In adult trials, metformin decreased the risk of myocardial infarction (39%), coronary deaths (50%), and all-cause mortality (36%) compared with standard therapy [27]. Moreover, in adults, metformin is also assumed to have positive effects in reducing the risk of developing diabetes mellitus among individuals at risk for the disease. The findings suggest that metformin might potentially be a valuable intervention for diabetes prevention, particularly in high-risk populations. However, this indication is considered as off-label use [28,29]. Finally, it is worth mentioning that metformin does not increase the risk of hypoglycemia during therapy [23,30].

Metformin is typically prescribed at daily doses ranging from 500 to 2000 mg divided twice daily. Generally, this oral medication is safe and, in most cases, well tolerated. The main adverse effects include nausea, vomiting, abdominal bloating, diarrhea/constipation, weakness, or a metallic taste in the mouth [6,22].

Metformin is not approved for weight loss in the pediatric population. However, it has been extensively used as an off-label for weight management in young individuals with excessive weight [31]. In some controlled trials among children with obesity, metformin demonstrated a favorable short-term effect on weight reduction and improvement of insulin sensitivity compared with a placebo and lifestyle modification [32]. However, there are limited studies on the treatment of T2D in children and adolescents. The largest clinical trial, the TODAY study, showed that metformin in monotherapy failed in almost 52% of children with T2D, while adjuncts to rosiglitazone (the thiazolidinedione class) and lifestyle intervention failed in 38.6% and 46.6%, respectively [12,14,33,34]. It suggests that metformin is insufficient to produce durable glycemic control and, therefore, is not an optimal treatment option in youth with T2D. The management of childhood-onset T2D must be optimized and should consider not only pharmacologic regimens but also lifestyle modification, along with close monitoring and follow-up. Notably, metformin was reported to have a much higher failure rate in adolescents compared to adults treated with metformin alone [6,7,12,13,14].

### 3.3. Insulin

In children and adolescents with diabetic ketoacidosis or HbA1c ≥ 8.5% (69 mmol/mol), insulin treatment is an alternative for initial management. There are several different insulin regimens. However, an initial dosing of 0.25–0.5 units/kg of basal insulin is usually effective in achieving good T2D control [6,7]. The main drawback of insulin treatment is weight gain, which results mainly from an increased appetite, insulin’s anabolic effect, and lowering glucose levels under the renal threshold [35]. Insulin-induced weight gain may negatively impact recommended management. Therefore, patients may experience dissatisfaction, which can cause treatment discontinuation or decreased adherence [36]. Another issue that might occur during insulin therapy is hypoglycemia, which is not very common in youth with T2D because of observed insulin resistance [6]. After treating acidosis, metformin should be started and slowly titrated. Transition to metformin takes approximately 2 to 6 weeks by reducing the total daily dose of insulin by around 30–50% with a parallel increase in metformin dosing [6,7]. Data from the TODAY study showed that, in most cases, insulin can be eliminated from the regimen without losing glycemic control [37,38].

### 3.4. Sodium-Glucose Transporter 2 (SGLT2) Inhibitors

Yet, in the pediatric population, there are limited therapeutic options for treating T2D compared to adults. Therefore, subsequent approvals of newer agents used in childhood-onset T2D treatment are promising and extend potential health benefits to patients facing difficulty controlling the disease. Recently, the FDA expanded the indication of two SGLT2 inhibitors (empagliflozin and dapagliflozin) to include children aged ≥10 years with T2D combined with diet and exercise to attain better metabolic control. These antidiabetic drugs act by inhibiting renal SGLT2, located in the proximal collecting tubule of the nephron. Then, SGLT2 inhibitors increase glucosuria, reducing blood glucose concentration and improving HbA1c. To date, this pharmacological class is assumed to significantly improve cardiovascular outcomes among high-risk patients and slow the progress of kidney disease in individuals with T2D. Beyond benefits, SGLT2 inhibitors also are related to some side effects. The most common adverse events are female genital mycotic infections, urinary tract infections, increased urination, nausea, and constipation. However, it might also be associated with some severe effects, such as volume depletion, acute kidney injury, hypoglycemia, or diabetic ketoacidosis (DKA) [39,40].

Previously, empagliflozin was tested in one phase trial assessing the pharmacokinetic and pharmacodynamic characteristics in adolescents with T2D to identify the appropriate doses for further pediatric use. The main outcome of this study confirmed that the pharmacokinetic profile of the 10 and 25 mg doses of empagliflozin already used in adult-onset T2D is similar in youth with T2D [41]. However, empagliflozin received a positive opinion (June 2023) based on the phase 3 DINAMO trial. This double-blind, placebo-controlled trial included 157 patients between 12 and 17 years of age with uncontrolled T2D (HbA1c ranging between 6.5 and 10.5% (mmol/mol) [42,43,44,45,46,47,48,49,50,51,52,53,54,55,56,57,58,59,60,61,62,63,64,65,66,67,68,69,70,71,72,73,74,75,76,77,78,79,80,81,82,83,84] initially taking metformin and/or insulin. Participants were randomly assigned to three groups, receiving empagliflozin 10 mg, linagliptin 5 mg (dipeptidyl peptidase-4 [DPP-4)] inhibitor), or placebo orally once a day for 26 weeks. At week 12, individuals in the empagliflozin group who did not experience a lowering of HbA1c to less than 7.0% (<53 mmol/mol) were again randomized at week 14 to remain on 10 mg dose or increased to 25 mg. The primary endpoint was a significant reduction in HbA1c level from the beginning of the on-treatment period to the end (26 weeks) compared with the placebo. This study demonstrated that empagliflozin as an adjunct to other treatment methods (diet, exercise, metformin, and/or insulin) significantly reduced HbA1c by 0.8% at week 26 compared with the placebo. Meanwhile, those on linagliptin did not experience statistical significance in HbA1c decrease compared to the placebo. When it comes to the overall safety profile of empagliflozin, it is comparable to the profile observed in adults. The most-reported side effect was hypoglycemia, followed by urinary tract infections. No cases of DKA or necrotizing fasciitis were reported [85].

The second SGLT2 inhibitor, dapagliflozin, was approved by the FDA in June 2024. The approval was supported by the results from the T2NOW phase 3 trial. In the trial, children between 10 and 17 years of age with insufficient control of T2D (HbA1c ranging between 6.5 and 10.5% (mmol/mol) [42,43,44,45,46,47,48,49,50,51,52,53,54,55,56,57,58,59,60,61,62,63,64,65,66,67,68,69,70,71,72,73,74,75,76,77,78,79,80,81,82,83,84] already on metformin, insulin, or both were randomized to receive 5 mg dapagliflozin, 2.5 mg saxagliptin (dipeptidyl peptidase-4 [DPP-4)] inhibitor), or placebo as an add-on treatment for 26 weeks. Participants in active treatment groups who did not attain HbA1c less than 7% (<53 mmol/mol) at week 12 were again randomly assigned to continue the dose or to receive an increased dose (10 mg of dapagliflozin or 5 mg of saxagliptin). Data from the T2NOW phase 3 trial confirmed that dapagliflozin is related to significant improvement in metabolic control in children and adolescents with T2D. At 26 weeks, participants assigned to dapagliflozin had a 0.62% point reduction in HbA1c compared with a 0.41% point increase for the placebo group. Moreover, subanalysis has presented a 1.1% HbA1c drop in adolescents who reported consistent use of dapagliflozin [86]. Safety outcomes in the T2NOW phase 3 trial study aligned with those reported in adults with T2D. The most common adverse effect was headache, followed by hypoglycemia [87].

Apart from the beneficial effect on glycemia control in individuals with T2D, as it has been demonstrated in adult studies receiving SGLT2 inhibitors, those drugs are related to a reduction in cardiovascular disease risk by decreasing blood pressure and increasing HDL cholesterol [47]. Then, in adults, SGLT2 inhibitors have a wider indication profile compared to youth. These agents may be indicated for adults with TD2 and established cardiovascular disease to reduce the risk of major adverse cardiovascular events. Moreover, SGLT2 inhibitors are also indicated for adults with heart failure, particularly those with reduced ejection fraction (HFrEF), regardless of the presence of diabetes, to reduce the risk of hospitalization and cardiovascular death or for those with chronic kidney (CKD) disease at risk of progression [42,88].

### 3.5. Glucagon-like Peptide-1 (GLP-1) Receptor Agonists

Glucagon-like peptide-1 (GLP-1) agonists represent a group of medications primarily aimed at treating T2D; however, they are now more widely used in children and adolescents with obesity. GLP-1, an incretin hormone, is produced mainly by enteroendocrine L cells in the distal ileum and colon, alpha cells of the pancreatic islets, and hindbrain neurons in the central nervous system. GLP-1 agonists stimulate insulin secretion from pancreatic beta-cells in response to carbohydrates absorbed from the gut. Then, the main function of GLP-1 is thought to be enhancing postprandial insulin release. Moreover, GLP-1 agonists slow gastric emptying, protect beta-cells of the pancreatic islets against the inflammation and apoptosis caused by cytokines, and inhibit hepatic gluconeogenesis. These drugs also improve insulin sensitivity through two potential mechanisms: directly augmenting glucose uptake by peripheral tissues such as skeletal muscles and indirectly promoting weight reduction [43]. A beneficial effect of these agents on weight loss is mainly related to suppressed gastric emptying, which stimulates satiety and appetite reduction at the level of the hypothalamus. Symptoms of delayed gastric emptying, including nausea and vomiting, are the main side effects caused by GLP-1 agonists.

Moreover, GLP-1 agonists in adult population studies are well documented to have a similar magnitude of effect on major adverse cardiovascular events, all-cause mortality, and cardiovascular-related death in overweight/obese individuals with and without T2D [44,45]. Yet, while GLP-1 agonists have been approved for use in children with T2D aged 10 and older for the treatment of obesity, their cardiovascular benefits have not been established in pediatric populations to the same extent as in adults [46].

Numerous studies also suggested that GLP-1 agonists generally have a positive effect on blood lipid profiles [47]. For instance, liraglutide combined with metformin decreased levels of cholesterol and LDL cholesterol in adult patients with T2D and cardiovascular disease already taking statins [48]. Furthermore, the specified analysis revealed that liraglutide significantly lowers blood concentrations of multiple lipid species, including ceramides, phosphatidylcholines, phosphatidylethanolamines, and triglycerides [49]. Some studies also suggested that GLP-1 agonists may improve the HDL cholesterol profile, although the results can vary depending on the specific agent and individual patient factors [50,51]. The exact mechanisms by which GLP-1 agonists affect lipid levels are still being studied, but they may involve effects on hepatic fat metabolism, appetite regulation, and overall weight reduction.

Finally, it must be highlighted, besides several beneficial effects, that these medications might increase the risk of thyroid cancer. The potential link between the new onset of thyroid cancer and GLP-1 agonist use is not yet fully explored. Firstly, this issue was highlighted in the premarketing phase after animal studies demonstrated raised rates of thyroid C-cell tumors in rodents [52]. GLP-1 receptor agonists receptors are expressed in different organs, including the lung, kidney, stomach, heart, and intestine; α, β, and d cells of the pancreatic islets; and multiple regions of the CNS. Also, this receptor might be widely expressed on the parafollicular C-cell membrane. After binding to the receptor, native GLP-1 stimulates cell proliferation, creating a favorable environment for tumor development [53]. Chronic stimulation of C-cells may lead to overproduction of calcitonin in a cAMP-dependent manner [54]. Therefore, this is a possible mechanism in which GLP-1 may promote rodent thyroid carcinogenesis, but there is insufficient evidence indicating the potential cancerogenic effect in humans [52]. Nevertheless, *GLP-1 cannot be used in individuals with their own or family medical history of medullary thyroid carcinoma or patients with multiple endocrine neoplasia syndrome type 2* [5,17,55].

Liraglutide in a dose of 0.6–1.8 mg/daily (Victoza) is indicated in pediatric patients ≥ 10 years with uncontrolled T2D as additions to lifestyle modification and oral metformin to attain sufficient metabolic control of the disease. The efficacy of liraglutide in youth-onset T2D was widely studied in the Evaluation of Liraglutide in Pediatrics with Diabetes (Ellipse) trial. The Ellipse study was a double-blinded trial in which 135 participants between 10 and 17 years with T2D were enrolled and randomly assigned to receive liraglutide or placebo for a 26-week period, followed by a 26-week open-label extension. Compared to the placebo, this study demonstrated superiority in improving metabolic control in the liraglutide group. In detail, at week 26, the mean HbA1c level decreased to 0.64% from the beginning of the study among those on liraglutide and only 0.42% in the placebo group. Furthermore, at week 52, the drop in HbA1c concentration was even more significant in the liraglutide patients than in placebo. Regarding the beneficial effect of GLP-1 agonist on weight loss, in the first part of the trial, there was no difference in BMI between the two groups of participants; however, by week 52, individuals on liraglutide experienced a reduction in BMI [56]. Then, adding liraglutide to therapy seems to be a relevant and promising treatment option for youth with obesity-induced T2D. Nevertheless, when choosing a second-line drug, one should consider the glycemic target, dosage schedule, route of administration, impact on weight, adverse effects, and possible comorbidities.

A higher dose of liraglutide (3 mg a day, Saxenda) is recommended in children ≥12 years of age with obesity defined as a BMI ≥ 30 kg/m^2^ or if ≥27 kg/m^2^ in the presence of one or more comorbidities for weight management. Recent clinical trials showed that liraglutide 3.0 mg yielded significant weight loss when combined with lifestyle intervention. In a double-blind study, a total of 251 youth between 12 and 17 years of age with obesity and insufficient weight management were randomized to receive 3.0 mg of subcutaneous liraglutide once a day (125 adolescents) for 26 weeks, followed by a 26-week observational period or a placebo in addition to lifestyle intervention for a 56-week treatment period. In this study, participants in the liraglutide group had a greater decrease in BMI compared to individuals assigned to the placebo group (43.3% vs. 18.7%, respectively). Nonetheless, after treatment withdrawal in youth on liraglutide, a greater rise in BMI was noticed compared to placebo. It suggests that young patients with obesity should receive a solid education on lifestyle modifications, which must be repeated to avoid the child’s demotivation regarding eating and physical activity habits. Also, it highlights the importance of introducing new pharmacological agents to weight management because, in most cases, lifestyle intervention is not enough to achieve set goals. Available data suggest that liraglutide 3.0 mg significantly affects weight reduction. However, individuals receiving liraglutide experienced more frequent gastrointestinal side effects than those in the placebo group. Then, adverse effects of GLP-1 were associated with treatment discontinuation in 10.4% of participants in this study [57]. Nevertheless, liraglutide 3.0 mg seems to be a reasonable option for the treatment of youth with obesity.

In 2021, medicine agencies for the drug administration (FDA and EMA) gave a positive opinion on an extended-release version of the GLP-1 drug, exenatide (BYDUREON BCise); since then, this agent has also been recommended in children and adolescents aged 10 to 17 years old with T2D in conjunction with nutritional management and exercise to achieve and maintain good metabolic control. The new approval for exenatide follows results from a clinical trial called BCB114, in which the safety of BYDUREON BCise was investigated among young people aged 10–17 years old. This study demonstrated that exenatide has a greater effect on HbA1c level reduction than the placebo [58].

In some countries, dulaglutide (Trulicity) is also available, which is approved for youth aged 10 years or older who suffer from T2D, in addition to lifestyle modification. In the AWARD-PEDS trial, treatment with dulaglutide agonist was associated with significant decreases in HbA1c levels compared to placebo among youth aged 10–17 years. This study included 154 individuals aged 10–17 with T2D taking metformin alone or without insulin or lifestyle modifications. Individuals were randomly assigned to three groups receiving a placebo, a 0.75 mg dose of dulaglutide, or a 1.5 mg dose of dulaglutide. Findings from this study showed that dulaglutide injected once a week is significantly more effective in achieving good metabolic control in adolescents with T2D than placebo. Over 26 weeks of treatment, HbA1c and fasting glucose levels were greatly reduced in the dulaglutide groups. However, there was no beneficial effect on BMI reduction [59].

Finally, semaglutide (Wegovy) was approved to manage childhood obesity in individuals aged 12 years and older [17,60]. The new indication for semaglutide follows results from the STEP TEENS clinical trial. This phase 3 study included 201 teens aged between 12 and 17 years, with obesity or overweight and at least one obesity-associated comorbidity. Participants were randomly selected to receive once-weekly semaglutide 2.4 mg injections or placebo in conjunction with diet and physical activity modifications for a 68-week period. This on-treatment phase was followed by a 7-week follow-up period during which the participants from both groups continued only lifestyle intervention. Efficacy endpoints were evaluated from the time of randomization to the end of the treatment period. The primary endpoint was the percentage change in BMI, and the secondary endpoint was a decrease in body weight of at least 5%. In the STEP TEENS clinical trial, semaglutide achieved superior mean percentage change in BMI in adolescents with obesity compared to the placebo group. The mean percentage change in BMI from the beginning to the end of the on-treatment phase was estimated at 16.1% with semaglutide and only 0.6% with placebo. Moreover, when participants were still on lifestyle interventions, the BMI stayed below the baseline level in individuals receiving semaglutide and above the baseline value in those assigned to a placebo. Similarly, body weight decrease from the study’s beginning to week 68 was significantly higher in the semaglutide group than in the placebo group. At the end of the treatment period, 73% of adolescents on semaglutide experienced at least a 5% reduction in their initial body weight. In contrast, for those who received a placebo, such reduction was only observed in 18% of the participants. The improvement in body weight resulted in a reduction in HbA1c, lipid profiles, and waist circumference. Therefore, semaglutide seems to decrease the risk of cardiovascular disease. Again, this study confirmed that GLP-1 agonists are an optimal treatment option for youth with obesity having a poor response to standard management [60].

GLP-1 receptor agonists undoubtedly benefit youth with morbid obesity and T2D. However, some concerns regarding the unintended side effects of these drugs need to be highlighted. Cooper et al. pointed out that inappropriate reductions in energy intake associated with GLP-1 during a child’s critical stage of growth and development might negatively impact their health later in life. Also, the authors suggested the possibility of abuse in individuals suffering from eating disorders, as well as youth involved in competitive sports such as gymnastics or ballet [61]. Finally, some postmarketing reports suggested the association between GLP-1 agonist use and suicidal thoughts, prompting the FDA to evaluate a causal relationship. However, after an investigation, the FDA did not find evidence that the use of these medicines causes suicidal thoughts or actions. So, implementing GLP-1 treatment should be carried out by an experienced clinical professional with further monitoring of adverse consequences for children’s health. Table 1 gives an overview of the effects and mechanism of action of antidiabetes drugs used recently in pediatric populations.

### 3.6. Challenges and Special Considerations of Youth-Onset T2D Management

Youth-onset T2D presents unique challenges and considerations both in research and clinical practice due to its distinct characteristics compared to adult onset. Scientific research on T2D is limited in the childhood population, and it is mostly related to difficulties in including appropriate participant numbers, stringent eligibility criteria, limited sites worldwide suitable for such studies, and the growing number of trials vying for the few eligible patients. In addition, research involving children and adolescents requires strict ethical oversight. Obtaining consent or assent from minors and managing potential risks are more complex than in adult populations [62,63,64]. These important concerns were highlighted by the SEARCH for Diabetes in Youth study, which pointed out that the time between diagnosis and enrollment in clinical trials for youth T2D was more than double that for children with T1D [65]. Therefore, medical knowledge on the mechanism of action, use indications, and beneficial or adverse effects are generally based on results obtained in studies on adults with T2D. In consequence, in adults, there is a wider range of medications that might be included in T2D management.

Generally, the mechanisms of action of antidiabetic agents are generally comparable across different age groups, including children, adolescents, and adults, as these drugs target specific biological pathways involved in glucose metabolism and insulin regulation. However, there are potential metabolic differences between adults and children, which can affect pharmacokinetics and pharmacodynamics. It might result from differences in body composition, organ function, and enzyme activity; therefore, it theoretically impacts the dosing, frequency, and efficacy of medications [66].

Another issue is the developmental consideration, which makes T2D treatment more complicated in children than adults. Puberty is a dynamic period of development marked by rapid changes in body size, shape, and composition, all of which might influence the treatment of T2D in children and adolescents. Moreover, youth have special nutritional requirements, making it fundamental to find a balance that avoids deficiencies and potential consequences like stunted growth while maintaining glycemic control [67,68].

Finally, adherence to T2D in youth might be lower compared to adults. Some studies have shown that adults with T2D do not have satisfactory levels of adherence to lifestyle modifications and/or medication regimens due to the complexity of treatment. Adolescents are undergoing significant physical and emotional changes, which can impact their ability to manage a chronic condition. Additionally, motivation to follow the medical recommendations might be lower in children; thus, youth need more support for motivation from parents or healthcare providers [69,70]. Still, data on treatment adherence in youth with T2D are scarce; however, some studies conducted in children with T1D showed that adherence to therapy declines over time [71]. Then, it might be assumed that youth with T2D can also be characterized by similar patterns of adherence.

### 3.7. Other Antiobesity Medications

#### 3.7.1. Orlistat

Orlistat (Xenical) is another agent that is used in some countries together with a healthy diet and exercises in youth older than 12 years with a BMI greater than 27 kg/m^2^ who have already developed hypertension, T2D, or hyperlipidemia [17]. This drug prevents the absorption of dietary fat by reversibly inactivating gastrointestinal and pancreatic lipases. The inhibition of lipases reduces the hydrolysis of triglycerides. Therefore, free fatty acids are not absorbed [72]. The effectiveness of orlistat was investigated in a double-blind study enrolling 539 individuals with excessive weight who were randomly assigned to the orlistat group or the placebo. After 54 weeks of treatment, a modest BMI decrease was observed in patients on orlistat compared to the control group. Interestingly, there were no significant changes in glucose concentration between the studied groups. Gastrointestinal side effects in those receiving orlistat were high at 50%, and most individuals had very low tolerability of the drug [73]. By inhibiting fat absorption, orlistat could be one of the promising drugs used for weight management, but the rate of adverse effects is relatively high, then it might be discouraging for the patients.

The adverse events of orlistat are closely related to its mechanism of action. Across the studies, the most frequently observed side effects include abdominal pain, fecal spotting, flatus with discharge, fecal urgency, and fatty/oily stool. However, individuals who start following a low-fat diet experienced less severe side effects. Therefore, orlistat might indirectly positively impact eating patterns. It should also be mentioned that patients on this treatment have a greater risk in developing vitamin deficiencies because orlistat affects the absorption of fat-soluble vitamins (A, D, E, and K) [74]. Multivitamin supplements, mainly vitamins A, D, E, and K, are recommended for patients taking orlistat. Finally, postmarketing reports of severe liver injury were reported. After evaluating the observed cases, the FDA did not find a causal relationship between orlistat and acute liver disease; however, relabeling the product and outlining the risks of potential liver injury with its use was recommended.

#### 3.7.2. Phentermine and Topiramate

Phentermine is one of the most recommended antiobesity drugs in adults, yet data on its effectiveness in youth are limited. This drug is an amphetamine derivative that inhibits simulation in the central nervous system. The main effect of phentermine is appetite reduction. Thereby, this medication contributes to weight loss [17,75,76]. In a retrospective medical chart review of children on a weight treatment program in an outpatient clinic, phentermine with lifestyle intervention was superior in weight reduction at one month, three months, and six months among youth with obesity relative to the control group. At three and six months, most participants receiving phentermine experienced a significant BMI reduction [76].

While topiramate is an antiepileptic typically used in adolescents with epilepsy and migraine, this drug also shows a profound effect on weight reduction. Some animal studies indicated that topiramate increases energy expenditure and reduces food intake, decreasing caloric intake. Still, the proper mechanism of topiramate on weight loss has yet to be fully understood [76,77]. In a double-blind trial among adults with obesity complicated by T2D, topiramate was associated with a great decrease in body weight and improvement in glucose profile (fasting and postprandial) and free fatty acid [78]. Recently, the combination of phentermine and extended-release topiramate (Qsymia) was included in weight treatment by the FDA in conjunction with diet and exercise in children ≥ 12 years of age with obesity. The pediatric indication was based on data from a 56-week, double-blind, placebo-controlled study conducted in the United States. In this study, 223 children with obesity between 12 and 16 years of age were selected to take mid-dose phentermine/topiramate 7.5 mg/46 mg or high-dose phentermine/topiramate 15 mg/92 mg or placebo as adjunct dietary restriction and exercise. After 56 weeks of treatment, the drop in BMI in individuals receiving high-dose phentermine/topiramate was more significant than in those taking placebo and mid-dose phentermine/topiramate [79]. Therefore, combining phentermine and extended-release topiramate could be a good option for supporting weight reduction in young individuals with obesity.

The side effects observed in the pediatric population while taking phentermine/topiramate are generally in line with those reported in adult trials testing this agent. The occurrence of adverse drug reactions of phentermine/topiramate depends on its dose. In accordance, treatment side effects were reported in 37% and 52.2% of patients in phentermine/topiramate 7.5 mg/46 mg and 15 mg/92 mg, respectively. The most common adverse events related to phentermine/topiramate are nausea, pyrexia, dizziness, arthralgia, influenza, ligament sprain, and depression [79]. The results of the included evidence-based studies regarding new-generation drugs used in childhood obesity and T2D management are demonstrated in Table 2 and Table 3. 

The characteristics of the most recent pharmacologic agents approved for treating obesity and T2D in youth are summarized in Table 4.

### 3.8. Bariatric Surgery

In children experiencing obesity and its health consequences, weight-loss surgery was proven to be a safe and efficient option to manage weight. Nowadays, bariatric surgery is one of the most effective approaches for substantial and persistent weight reduction and improving coexisting complications. However, the few existing guidelines on bariatric surgery for children and adolescents give somewhat different recommendations. In 2015, the European Society for Paediatric Gastroenterology, Hepatology, and Nutrition (ESPGHAN) advised bariatric surgery as an option for adolescents who had achieved 95% of the expected height and a BMI ≥ 40 kg/m^2^ with severe comorbidities and BMI ≥ 50 kg/m^2^ with mild comorbidities [80]. According to the latest evidence on weight management and prevention of cardiovascular risk factors, the American Society for Metabolic and Bariatric Surgery (ASMBS) and the American Academy of Pediatrics (AAP) produced a consensus that recommends antiobesity surgery in children and adolescents who suffer from moderate obesity (BMI of 35 kg/m^2^ or higher) with significant health complications related to obesity or severe obesity (40 kg/m^2^) [4,81]. However, the decision regarding bariatric surgery is typically a multidisciplinary process and involves various healthcare professionals to ensure comprehensive evaluation and care for the patient. Usually, this multidisciplinary team includes pediatricians, endocrinologists, pediatric surgeons, dietitians, psychologists, and psychiatrists. The team ensures that the child meets specific criteria, including age, weight, health status, and psychosocial factors. Parental consent and involvement in the decision-making process are also crucial. The ultimate goal is to ensure that the benefits of the surgery outweigh the risks and that it is in the best interest of the child’s long-term health [5,80,81].

Currently, antiobesity surgery is related to substantial body mass reduction and remission of T2D in youth. In pediatric populations, two main types of weight-loss surgery are commonly recommended: the laparoscopic sleeve gastrectomy and the Roux-en-Y gastric bypass. The largest study testing bariatric surgery’s effectiveness in youth, the Teen Longitudinal Assessment of Bariatric Surgery (Teen-LABS), showed sustainable effects on weight reduction, improved cardiometabolic health, and slowed disease progress. The Teen-LABS study is the first trial, which included 242 youth with obesity who underwent Roux-en-Y gastric bypass or sleeve gastrectomy between 2007 and 2012. At the beginning of the study, the mean age of participants was 17 years, and the initial BMI was estimated at around 53 kg/m^2^. Three years after the bariatric procedure, the body mass, the appearance of obesity-related conditions, and other risk factors of cardiometabolic diseases were evaluated. At the end of the follow-up period, a significant weight reduction was observed in both groups. However, the two procedures performed had no difference in weight loss. Then, this study showed that Roux-en-Y gastric bypass and sleeve gastrectomy have similar efficacy in treating children and adolescents with obesity. In addition, coexisting conditions such as dyslipidemia, hypertension, and T2D were significantly decreased or even in remission within three years [89]. Finally, compared to the outcomes of two large studies in adolescents with obesity, the TODAY study (intensive nonsurgical treatment) vs. the Teen-LABS study, bariatric surgery was significantly more effective than pharmacological management. Adolescents with T2D who underwent bariatric surgery experienced a greater decrease in BMI than those matched by baseline age (13–18 years), race, sex, ethnicity, and baseline BMI (>35 kg/m^2^) medical controls from the TODAY study (29% vs. 3.7%). Also, participants from the Teen-LABS study had a greater reduction in the average HbA1c levels. In detail, HbA1c concentration declined from 6.8 to 5.5% in Teen-LABS participants, while in the TODAY group, HbA1c rose from 6.4 to 7.8% during two years [83]. Finally, it seems that having earlier bariatric surgery might have better results than waiting until adulthood. Five years after surgical intervention, adolescents had greater results in maintaining weight and treating other weight-related conditions than adults receiving the same procedure in the LABS (Longitudinal Assessment of Bariatric Surgery) study. Most adults from the LABS study already suffered from obesity in childhood but did not receive surgery until they were adults [84]. Nutritional deficiencies are the main concern in developing adolescents who receive surgical treatment. Over the period of 2 years after surgery, low ferritin levels were found in 57%, while deficiency of B12 or any other vitamins was reported in 35% and 16% of participants, respectively [85]. Therefore, after bariatric surgery, most adolescents will require vitamin supplementation. Moreover, bariatric surgery in adolescents might develop other long-term postoperative complications. The most observed complications include gastroesophageal reflux disease (GERD), hiatal hernia, and treatment failure requiring operative revision. Less common adverse effects are liver necrosis, gallbladder disorders, pancreatic disorders, acute kidney failure, and neuromuscular or skin complications. Finally, GERD and hiatal hernia may lead to Barrett’s esophagus; therefore, youth after bariatric surgery are at risk in developing esophageal adenocarcinoma [89].

## 4. Strengths and Limitations of the Review

The strength of the present narrative review is that it provides a practical summarization of the available knowledge on the most recent therapeutic options for treating adolescents with obesity and T2D. In detail, this work discusses the most significant trials assessing the efficacy and effectiveness of the lately approved agents. It also provides the indication for each drug and evaluates its potential benefits as well as adverse effects. Thus, it gives a broader perspective and hints at the possible treatment methods in the pediatric population affected by obesity and T2D. Mainly, it might be valuable for everyday clinical practice. However, concerning the limitations of our study, this work is not a systematic review. It may not cover all relevant issues or include every pertinent study, potentially leaving out some aspects of the complex management of obesity and T2D in youth. The review’s findings are based on selected literature and expert opinion, which may not fully represent the breadth of evidence available in comprehensive guidelines. Consequently, while the review provides valuable insights, it should be considered alongside more exhaustive systematic reviews and clinical practice standards for a complete understanding of childhood obesity and T2D management.

## 5. Conclusions

The incidence of T2D in the pediatric population is rising, driven by increasing levels of excess weight and obesity. Youth-onset T2D is characterized by lower insulin sensitivity, insulin hypersecretion, and rapid deterioration in beta-cell function compared to adult-onset T2D. In consequence, children and adolescents with early-onset T2D have a more rapid progression of microvascular and cardiovascular disease than young individuals with type 1 diabetes and those with adult-onset T2D. Unfortunately, the benefits of lifestyle interventions observed in clinical trials might be challenging to translate to real-world settings, and most young people do not achieve set goals in everyday clinical practice. Early recognition and management of childhood obesity should aim at the prevention of its comorbidities, including T2D. A multifaceted approach to childhood obesity and T2D is essential, and young people should receive intensive treatment as early as possible, including the use of newer pharmacological agents in conjunction with behavior and lifestyle modifications. Nevertheless, when choosing a second-line drug, one should consider the glycemic target, dosage schedule, route of administration, impact on weight, adverse effects, and possible comorbidities. In certain circumstances, treatment might include weight-loss surgery. In particular, antiobesity surgery is related to significant weight reduction, remission of T2D, and lower overall mortality in youth.

## Figures and Tables

**Table 1:** Overview of effects and mechanisms of action of approved antidiabetic agents in youth [22,23,24,25,26,27,28,29,37,38,40,41,42,43,44,45,46,47,52,85,86,87,88].

	Biguanides (Metformin)	SGLT2 Receptor Agonists (Empagliflozin/Dapagliflozin)	GLP-1 Agonists (Liraglutide/Exenatide)
Effects/Mechanism of actions	Acts via AMP kinase in liver, muscle, and fat. Inhibit hepatic gluconeogenesis and increase peripheral glucose uptake and insulin sensitivity.	Inhibit renal tubular reabsorption of glucose and lower the renal threshold for glucose, thereby increasing urinary glucose excretion.	Increase insulin secretion proportionate to blood glucose concentrations, suppressing glucagon, prolonging gastric emptying, and promoting satiety.
Percent HbA1c lowering	1–2%	1–2% Dapagliflozin use in youth with T2D did not show benefit relative to metformin ± insulin, although subanalysis presented a 1.1% HbA1c drop in adolescents that reported consistent use.	Ellipse trial showed the liraglutide group had 1% and 1.5% HbA1c reduction at 26 and 52 weeks, respectively. Exenetide (Bydureon) 2 mg once a week lowered HbA1c by 0.85% compared to a placebo.
Cardiovascular benefits and risk	Reduce MI by 39% and coronary deaths by 50%.	Positive CV effect due to reduction in Na and UA absorption and reduction in BP.	Reduce CV risk.
Effect on lipid profile			
HDL cholesterol level	↑	↑	↑
LDL cholesterol level	↓	↔ or ↑	↓
Triglycerides level	↓	↔	↓
Weight loss	↔ or ↓	↓	↓↓
Risk of hypoglycemia	↔	↔	↔

Abbreviations: SGLT2, sodium-glucose transporter 2 inhibitors; GLP-1, glucagon-like peptide-1; AMP kinase, Adenosine monophosphate-activated protein kinase; HbA1c, glycated hemoglobin; T2D, type 2 diabetes; MI, myocardial infarction; CV, cardiovascular; Na, sodium; UA, uric acid; HDL, high-density lipoprotein; LDL, low-density lipoprotein; BP, blood pressure; ↑, increasing trend; ↓, decreasing trend; ↓↓, strong decreasing trend; ↔, stable trend.

**Table 2:** Summary of included studies on T2D management in youth.

Study ID	Drug	Study Design	Endpoints	Outcome
Zeitler et al. [34]	Metformin (biguanides)	N = 699 youth with overweight and T2D receiving metformin (1000 mg b.i.d.) with HbA1c up to 8% aged 10–17 years Group 1: Metformin (1000 mg × 2/24 h) for 6 months Group 2: Metformin plus RZ (4 mg × 2/24 h) Group 3: Metformin plus lifestyle program	The primary endpoint was a loss of glycemic control, defined as HbA1c > 8% for 6 months or sustained metabolic decompensation requiring insulin	Metformin failed to control T2D in 51.7% of participants. Metformin plus RZ was more effective than metformin alone (51.7% vs. 38.6%). Metformin plus lifestyle intervention significantly differed from metformin alone or metformin plus RZ (46.6%).
Laffel et al. [37]	Empagliflozin (SGLT2 receptor agonists)	N = 157 adolescents with uncontrolled T2D (HbA1c ≥ 6.5 to ≤10.5%) despite metformin and/or insulin aged 10–17 years Group 1: Empagliflozin 10 mg (o.d.) for 26 weeks. At week 12, when HbA1c ≤ 7.0%, individuals were again randomized to remain on 10 mg or increase to 25 mg Group 2: Linagliptin 5 mg (o.d.) Group 3: placebo (o.d.)	The primary outcome was a change from baseline in HbA1c at 26 weeks	Empagliflozin as an adjunct to other treatment methods (diet, exercise, metformin, and/or insulin) significantly reduced HbA1c by 0.8% at week 26 compared with the placebo.
Shehadeh et al. [87]	Dapagliflozin (SGLT2 receptor agonists)	N = 256 adolescents with uncontrolled T2D (HbA1c ≤ 10.5%) despite diet and exercise and/or metformin and/or insulin aged 10–17 years Group 1: Dapagliflozin 5 mg (o.d.) for 26 weeks. At week 12, when HbA1c ≤ 7.0%, individuals were again randomized to remain on 5 mg or increase to 10 mg Group 2: Saxagliptin 2.5 mg (o.d.) for 26 weeks. At week 12, when HbA1c ≤ 7.0%, individuals were again randomized to remain on 2.5 mg or increase to 5 mg. Group 3: placebo for 26 weeks	The primary endpoint was a change in HbA1c at week 26	At 26 weeks, participants assigned to dapagliflozin had a 0.62% point reduction in HbA1c compared with a 0.41% point increase for the placebo group.
Tamborlane et al. [56]	Liraglutide (GLP-1 agonists)	N = 134 adolescents with BMI ≥ 85th percentile and uncontrolled T2D [HbA1c < 7.0 to ≤11.0% if participants treated with diet and exercise or HbA1c < 6.5 to ≤11.0% if they were on metformin (with or without insulin)] aged 10–17 years Group 1: S.C. liraglutide (dose increased 0.6, 0.9, 1.2, and 1.8 mg/24 h) for 26 weeks Group 2: placebo (26 weeks blinded and 26 weeks unblinded)	The primary outcome was a change from baseline in HbA1c at 26 weeks. Secondary endpoints included the change in FPG	At week 26, the mean HbA1c level decreased to 0.64% from a baseline in those on liraglutide and only 0.42% in the placebo group. FPG had reduced at both time points in the liraglutide group but had increased in the placebo group.
Tamborlane et al. [58]	Exenatide (an extended -release version of GLP-1 agonist)	N = 83 adolescents with uncontrolled T2D (HbA1c < 6.5 ≤ 11.0% if participants were not taking insulin, or HbA1c% < 6.5 ≤ 12.0% if they were insulin, or treated with diet and exercise or in combination with a stable dose of an oral antidiabetic drug and/or insulin for at least 2 months. Group 1: S.C. exenatide (2 mg q.w.) for 24 weeks Group 2: placebo for 24 weeks	The primary endpoint was a change in HbA1c at week 24	At 24 weeks, the least squares mean change in HbA1c was −0.36% for the exenatide and +0.49% for the placebo.
Arslanian et al. [59]	Dulaglutide (GLP-1 agonists)	N = 154 adolescents with uncontrolled T2D, HbA1c > 6.5% to <11%, treated with diet and exercise, with or without metformin and/or basal insulin, aged 10–17 years Group 1: S.C. dulaglutide (0.75 mg q.w.) for 26 weeks Group 2: S.C. dulaglutide (1.5 mg q.w) Group 3: placebo	The primary endpoint was the change from baseline in HbA1c level at 26 weeks; secondary endpoints included HbA1c < 7.0% and changes from baseline in the FPG and BMI	At 26 weeks, more participants on dulaglutide achieved HbA1c < 7% than in the placebo group (51% vs. 14%). FPG increased in the placebo group and decreased in the pooled dulaglutide groups, and there were no between-group differences in the change in BMI.

Abbreviations: T2D, type 2 diabetes; b.i.d., twice a day; HbA1c, glycated hemoglobin; RZ, rosiglitazone; SGLT2, sodium-glucose transporter 2 inhibitors; o.d., once a day; BMI, body mass index; GLP-1, glucagon-like peptide-1; S.C., subcutaneous; FPG, fasting plasma glucose; q.w., once a week.

**Table 3:** Summary of included studies of new agents for treating adolescents with obesity.

Study ID	Drug	Study Design	Endpoints	Outcome
Kelly et al. [57]	Liraglutide (GLP-1 agonists)	N = 251 adolescents with BMI ≥ 95th percentile and a poor response to lifestyle therapy aged 12–17 years Group 1: S.C. liraglutide (3 mg/24 h) for 26 weeks, followed by a 26-week observational period Group 2: placebo in addition to lifestyle intervention for a 56-week treatment period	The primary endpoint was the change from baseline in the BMI-SD at week 56	Participants on liraglutide had a greater decrease in BMI-SD than those from the placebo group (43.3% vs. 18.7%).
Weghuber et al. [60]	Semaglutide (GLP-1 agonists)	N = 201 adolescents with BMI ≥95th percentile or >85th percentile with 1 or more weight-related comorbidities: HA, dyslipidemia, obstructive sleep apnea, or T2D, and a poor response to lifestyle, aged 12–17 years Group 1: S.C. semaglutide (2.4 mg q.w.) for 68 weeks Group 2: placebo in conjunction with diet and physical activity modifications for a 68-week period	The primary endpoint was the percentage change in BMI from baseline to week 68; the secondary endpoint was a decrease in body weight of at least 5%	The mean change in BMI was −16.1% with semaglutide and 0.6% with placebo. At the end of the study, 73% of participants on semaglutide had a weight loss of 5% or more, as compared with 18% in the placebo group.
Chanoine et al. [73]	Orlistat (lipase inhibitor)	N = 593 adolescents with BMI ≥ 2 units above the 95th percentile, aged 12–16 years Group 1: a 120 mg dose of orlistat 3 times daily for 1 year, as an adjunct to a hypocaloric diet (30% fat calories) and lifestyle modifications Group 2: placebo for 1 year, as an adjunct to hypocaloric diet (30% fat calories) and lifestyle modifications	The primary endpoint was the percentage change in BMI from baseline to the end of the study; secondary measures included changes in WHR, weight loss, lipid measurements, and glucose and insulin responses to the OGTT	At the end of the study, BMI had decreased by 0.55 with orlistat but increased by 0.31 with placebo. WHR decreased in the orlistat group but increased in the placebo group.
Kelly et al. [79]	PHEN/TPM (anorectics/ anticonvulsants)	N = 223 adolescents with obesity, BMI ≥ 95th percentile, and a poor response to lifestyle therapy alone aged 12–16 Group 1: mid-dose PHEN/TPM (7.5 mg/46 mg, o.d.) plus lifestyle therapy for 56 weeks Group 2: top-dose PHEN/TPM (15 mg/92 mg, o.d.) plus lifestyle therapy for 56 weeks Group 3: placebo plus lifestyle therapy for 56 weeks	The primary endpoint was the mean percent change in BMI from baseline to week 56	The primary outcome of percent change in BMI at week 56 showed differences from placebo of −10.44 percentage points and −8.11 percentage points for the top and mid doses of PHEN/TPM, respectively.

Abbreviations: GLP-1, glucagon-like peptide-1; BMI, body mass index; BMI-SD, body mass index- standard deviation; HA, hypertension; T2D, type 2 diabetes; q.w., once a week; S.C., subcutaneous; WHR, waist-to-hip ratio; OGTT, oral glucose tolerance test; o.d., once a day; PHEN/TPM, phentermine/topiramate.

**Table 4:** Most recent pharmacologic agents approved for treating obesity and T2D in youth [34,35,38,39,40,41,47,85].

Medication	Indication	Benefits	Potential Side Effects	Approval FDA/EMA
**SGLT2 inhibitors (empagliflozin/dapagliflozin)**	As an adjunct to diet and exercise to improve glycemic control in children aged 10 years and older with T2D	Beneficial effect on HbA1c	UTI, female/male genital mycotic infections, URTIs, polyuria, back pain, nausea, dyslipidemia, increases serum creatinine and decreases eGFR, renal impairment, necrotizing fasciitis of the perineum, and DKA.	FDA/EMA
	Liraglutide (Victoza, 0.6–1.8 mg daily): T2D in children ≥ 10 years	Beneficial effect on HbA1c and weight loss, reduction in the risk of T2D complications	Gastrointestinal nausea, vomiting, and diarrhea. Hypoglycemia (S). Warnings: personal or family history of medullary thyroid carcinoma or in patients with MEN2, pregnancy.	FDA/EMA
**GLP-1 receptor** **agonist**	Liraglutide (Saxenda, 3 mg a day): weight management in adolescents ≥ 12 years	Beneficial effect on weight loss	Cautions: acute pancreatitis, acute events of gallbladder disease, and renal impairment related to dehydration.	
	Exenatide (BYDUREON BCise): T2D in children ≥ 10 years	Beneficial effect on HbA1c	The same as liraglutide plus injection site nodule.	
	Dulaglutide (Trulicity): T2D in children ≥ 10 years	Beneficial effect on glycemic control	The same as liraglutide plus DR progression among patients with a history of DR.	
	Semaglutide (Wegovy): weight management in adolescents ≥ 12 yrs	Beneficial effect on weight loss	The same as liraglutide plus hypoglycemia.	
**Orlistat** **(Xenical)**	Weight management for adolescents 12 years and older	Beneficial effect on weight loss	Oily spotting, abdominal pain, nausea, fatty/oil stool, reduced absorption of fat-soluble vitamins, and liver failure. Contraindications: chronic malabsorption syndrome, cholestasis. Cautions: If a meal is missed or contains no fat, the dose should be omitted, and a multivitamin supplement is recommended.	FDA
**Phentermine/** **topiramate (Qsymia)**	Weight management for adolescents 12 years and older	Beneficial effect on weight loss	Insomnia, dry mouth, increased heart rate, anxiety, increased blood pressure, cognitive dysfunction, metabolic acidosis, teratogenicity, and kidney stones. Contraindications: history of CVD, glaucoma, agitated states, hyperthyroidism, history of DA, use of MAOIs within the preceding 14 days.	FDA

Abbreviations: SGLT2, sodium-glucose transporter 2 inhibitors; T2D, type 2 diabetes; HbA1c, glycated hemoglobin; UTI, urinary tract infection; URTIs, upper respiratory tract infections; eGFR, estimated glomerular filtration rate; DKA, diabetic ketoacidosis; FDA, U.S. Food and Drug Administration; EMA, European Medicines Agency; GLP-1, glucagon-like peptide-1; MEN2, multiple endocrine neoplasia syndrome type 2; DR, diabetes retinopathy; CVD, cardiovascular disease; MAOIs, monoamine oxidase inhibitors; DA, drug abuse.

## Data Availability

No new data were created or analyzed in this study.
